# CoMATE-DETR: Coordinated Model Compression and Matching-Aware Training for Efficient Citrus Leaf Disease Detection

**DOI:** 10.3390/s26217088

**Published:** 2026-11-09

**Authors:** Chengyuan Shi, Na Yao

**Affiliations:** 1 College of Information Engineering, Tarim University, Alar 843300, China; 2 Key Laboratory of Tarim Oasis Agriculture, Ministry of Education, Tarim University, Alar 843300, China; 3 College of Cyberspace Security, Tarim University, Alar 843300, China

**Keywords:** citrus leaf disease detection, CoMATE-DETR, lightweight object detection, matching-aware training, RGB imaging, smart agriculture

## Abstract

Accurate citrus leaf disease detection requires a balance between visual discrimination and computational efficiency for camera-based monitoring in digital agriculture. This study proposes CoMATE-DETR, an efficient RT-DETR-based detector that coordinates model compression with matching-aware training. The framework integrates a partial-convolution backbone, external attention, a Dense O2O Matching Strategy (DOMS), and Matchability-Aware Loss (MAL) without adding inference branches. Experiments used five citrus leaf categories. Across training seeds 0, 1, and 2, the complete model achieved 91.70 ± 0.41% mAP@50:95 on the fixed 566-entry test evaluation, compared with 91.79 ± 0.41% for the compatible baseline. The reported architecture profile contains 9.7 M parameters and 17.6 GFLOPs, reductions of 51.7% and 41.7% from the baseline, with a throughput of 106 FPS on an NVIDIA GeForce RTX 4090. Factorial ablations increased mAP@50:95 from 90.56% for PConv + EA to 91.70% for the complete training configuration. PConv + EA + MAL achieved the highest ablation mean of 92.94 ± 0.89%. These results establish a compact detector with high detection accuracy and quantify the distinct effects of DOMS and MAL on the accuracy–efficiency balance. On the external BDLemonLeaf V6 Greening subset, the frozen checkpoint achieved 63.05% AP50:95, 95.32% AP50, and 77.67% AP75.

## 1. Introduction

Citrus disease monitoring depends on recognizing foliar symptoms that can inform scouting and subsequent diagnosis. Black spot, canker, greening, and melanose are relevant targets because infection can affect plant vigor, fruit quality, and marketable yield. However, symptom appearance varies with disease stage, cultivar, illumination, viewing distance, and background. This variation complicates visual assessment and makes reliable image screening a useful objective for camera-based monitoring.

Conventional scouting relies on repeated visual inspection by trained personnel, which is labor-intensive and difficult to standardize across large orchards. Camera-based sensing and deep learning offer a route to repeatable image screening [1]. Existing citrus datasets and recognition methods establish the feasibility of automated visual analysis [2,3,4]. Real-time detectors such as YOLO [5] extend this approach to localization, but their usefulness also depends on model size, computation, and processing delay.

Previous citrus studies show that image-analysis performance depends on the sensing system as well as the detector. Da Silva et al. [6] combined mobile edge AI with disease detection and orchard mapping. Huang et al. [7] integrated UAV image acquisition, object detection, and embedded computing for citrus fruit detection. Apacionado and Ahamed [8] used a low-cost surveillance camera to detect sooty mold at canopy level. Lee et al. [9] developed a web-based classification service using field-collected citrus pest images. Together, these studies establish practical routes for camera-based analysis, but their differing acquisition domains and task definitions limit direct performance comparisons.

Recent horticultural studies have adapted RT-DETR to reduce computation while retaining detection accuracy. Yuan et al. [10] combined an efficiency-oriented backbone, adaptive scale fusion, pruning, and edge-device evaluation for pomegranate detection. Shi et al. [11] developed a partial-convolution-based RT-DETR variant for blueberry maturity detection and reported inference on an embedded Jetson platform. A watermelon study examined small-object, occlusion, and low-light subsets, while identifying broader field trials and end-to-end latency measurements as further needs [12]. These studies support task-specific detector design and distinguish image-domain performance from evidence of deployment readiness.

Transformer detectors have evolved from DETR [13] and Deformable DETR [14] to RT-DETR [15], which combines multi-scale features with global context modeling. RT-DETR also avoids non-maximum suppression, making it relevant to sensing applications constrained by processing delay. For citrus leaf analysis, the unresolved design question is how far computation can be reduced without losing discriminative visual information. Small, blurred, or low-contrast leaf regions make this trade-off particularly important.

Recent real-time detectors address efficiency through both architectural design and training supervision. YOLOX [16] combines anchor-free prediction with separate classification and localization branches and the SimOTA assignment strategy. YOLOv9 [17] introduces programmable gradient information and a generalized efficient layer aggregation network to improve information preservation and parameter utilization. YOLOv10 [18] further combines consistent dual assignments for training without non-maximum suppression with an efficiency-oriented redesign of network components. RTMDet [19] examines the balance between backbone and neck capacity and introduces soft labels into dynamic assignment costs. These approaches indicate that an efficient detector cannot be characterized by parameter count alone: feature processing, assignment, and prediction design all affect its accuracy–cost balance. Their published benchmark results nevertheless depend on their respective training and inference protocols.

Transformer-based detection offers a complementary route through set prediction. DETR [13] and Deformable DETR [14] established a framework in which object queries predict categories and locations through one-to-one matching. RT-DETR [15] makes this framework more suitable for real-time processing by separating intra-scale interaction from cross-scale fusion in a hybrid encoder and improving the initialization of decoder queries. Its decoder retains prediction heads for category scores and bounding boxes. This structure provides an appropriate starting point for investigating compression, but reducing the backbone and encoder may alter the features available to the decoder. The accuracy consequences of those changes therefore require explicit ablation rather than an assumption that a smaller network preserves the original detector’s behavior.

Efficient operators and compact attention address different sources of inference cost. Chen et al. [20] proposed partial convolution in FasterNet to reduce redundant spatial computation and memory access by processing only part of the channel representation. Their analysis also distinguishes a reduction in operation count from a reduction in measured latency, which can depend on operator implementation and hardware utilization. Guo et al. [21] proposed external attention using learnable key and value memories shared across inputs. With a fixed memory size, its attention computation grows linearly with the number of input tokens. Combining these ideas motivates separate treatment of spatial feature extraction and context aggregation. The complete detector’s speed and accuracy are therefore reported after integration in Table 1 and Table 2.

Training supervision is another relevant consideration when reducing network capacity. DEIM [22] increases supervision density by constructing training images with more targets while preserving one-to-one assignment for each target. Its Matchability-Aware Loss uses localization quality to guide classification supervision and addresses the optimization of low-quality matches. These mechanisms operate during training and do not require additional inference branches. CoMATE-DETR integrates dense-target image composition and matching-aware classification supervision, and the factorial ablations in Section 3.1 evaluate DOMS and MAL as separate training factors.

Hybrid CNN–Transformer designs have also been investigated outside agricultural imaging. Maiza et al. [23] integrated an EfficientViT backbone with cascaded group attention into a YOLOv9-based detector for urban waste. Their work illustrates the use of local feature extraction and attention-based context modeling within a real-time detection system. Because its task, acquisition conditions, and evaluation protocol differ from citrus leaf disease detection, its reported performance is used as contextual reference rather than as a matched benchmark. Together with horticultural RT-DETR adaptations [10,11,12], this work motivates evaluation of task-specific architecture choices while keeping computational measurements and application-level validation distinct.

This study presents CoMATE-DETR for efficient citrus leaf disease detection through coordinated backbone compression, compact context aggregation, and matching-aware training. The study makes three contributions. First, it integrates partial convolution and external attention into an RT-DETR detector while retaining the standard decoder prediction heads. Second, it separates DOMS image composition from MAL classification supervision and quantifies their individual and combined effects across three training seeds. Third, it reports detection accuracy together with parameter count, operation count, and model-forward throughput, establishing the accuracy–efficiency profile of the compact detector.

## 2. Materials and Methods

### 2.1. Image Dataset and Split

**Figure 1:**
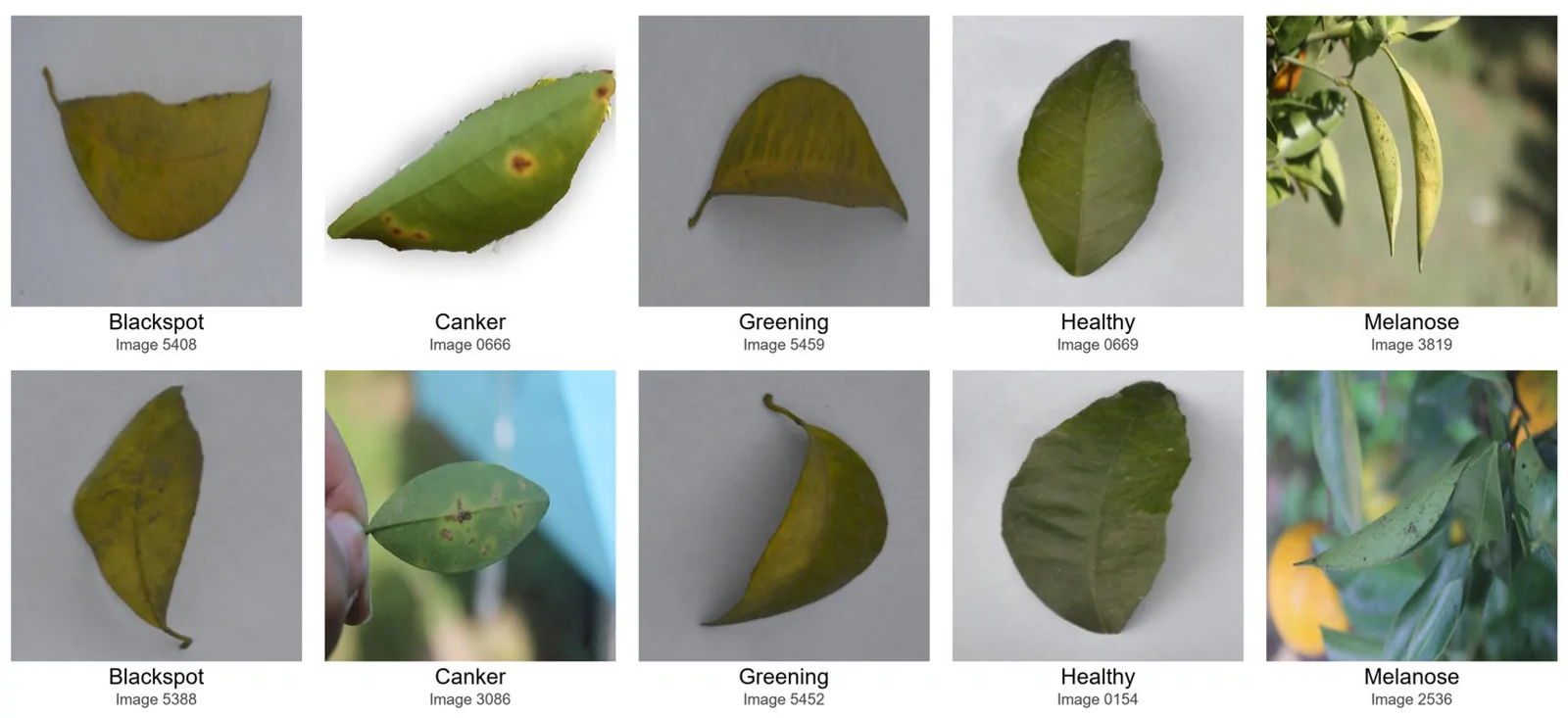
Example training images for the five dataset categories: Blackspot, Canker, Greening, Healthy, and Melanose. Each panel shows an unaltered image with its dataset class label and source filename identifier. Labels reproduce the supplied annotations.

This study uses the Jiangxi Citrus Leaf Disease Dataset described by Wu et al. [24]. The underlying records were supplied by a third-party data provider and obtained by the research team through an online data platform; the authors did not collect the images, and the dataset is not presented here as an official public benchmark. The images were collected primarily in citrus orchards in Ganzhou, Jiangxi Province, China, with a small supplementary set of web-sourced images. The dataset contains five categories: Blackspot, Canker, Greening, Melanose, and Healthy (Figure 1). The source study reports 5658 images at 640 × 640 resolution with an 8:1:1 split. The experimental split was fixed before model training, and its image lists and annotation inventory are summarized below.

The dataset contains 5658 image files across the training, validation, and test splits, with 4526, 566, and 566 entries, respectively. The audited annotations contain 12,771 valid bounding boxes: 10,165 in training, 1455 in validation, and 1151 in testing. Image counts refer to the file entries used in each split. Table A1 summarizes image and instance counts by object size, and Table A2 gives the class-wise distribution.

The validation split contains 566 images, and the test split contains 566 images; these fixed file lists were used for the corresponding evaluations. A review of the fixed image lists found no completely identical images shared between training and validation or between training and test. The available source records are image-level and do not include specimen-, plant-, or acquisition-session identifiers; split independence is therefore documented at the image-file level. The mean number of valid boxes per image is 2.25 for training, 2.57 for validation, and 2.03 for testing. Table A1 and Table A2 provide the corresponding object-size and class-wise distributions.

Healthy is class ID 3 in the five-category detection task. Healthy targets have instance bounding boxes and participate in both localization and classification; a single image can contain multiple Healthy instances. Object size is defined using bounding-box area *A* = *w* × *h* in the original image: small, 0 < *A* < 1024 pixels^2^; medium, 1024 ≤ *A* < 9216 pixels^2^; and large, *A* ≥ 9216 pixels^2^.

Annotation cleanup removed six zero-area Melanose box records with coordinates [314.25, 302, 0, 6] in [x, y, width, height] format: three in training, two in validation, and one in testing. The corresponding images and their other valid boxes were retained. Table A4 records the affected source files. The audited inventory contains no zero-area boxes, and every image retains valid annotations. Original annotations and the cleanup log are retained with the data package. Appendix A reports this cleaned inventory; Table 1, Table 2 and Table 3 retain the results of the completed model evaluations.

### 2.2. Image Preprocessing and Augmentation

Training augmentation uses a two-stage schedule. Before zero-based epoch 250, Mosaic is applied with probability 1.0. Four source images are resized to 640 × 640 and placed on a 1280 × 1280 canvas filled with RGB value 114; each mosaic center coordinate is sampled uniformly from 320 to 960. After panel placement and the final 640 × 640 canvas resize, bounding-box coordinates are multiplied by 0.5 and clipped to the output boundary, and non-positive boxes are removed. Mixup [25] is applied with a probability of 0.5 by cyclically shifting the batch by one position and blending each image with its shifted counterpart using a coefficient sampled uniformly from 0.45 to 0.55; the corresponding boxes and labels are concatenated. Random photometric distortion (*p* = 0.5), RandomZoomOut with fill value 0, RandomIoUCrop (*p* = 0.8, crop scale 0.3–1.0, aspect-ratio range 0.5–2.0, and 40 sampling trials), one-pixel bounding-box sanitization, horizontal flipping, image resizing, tensor conversion, and normalized cxcywh box encoding are applied in the configured pipeline. Image values are converted to floating-point tensors and scaled to [0, 1]; ImageNet mean/std normalization is not applied.

**Figure 2:**
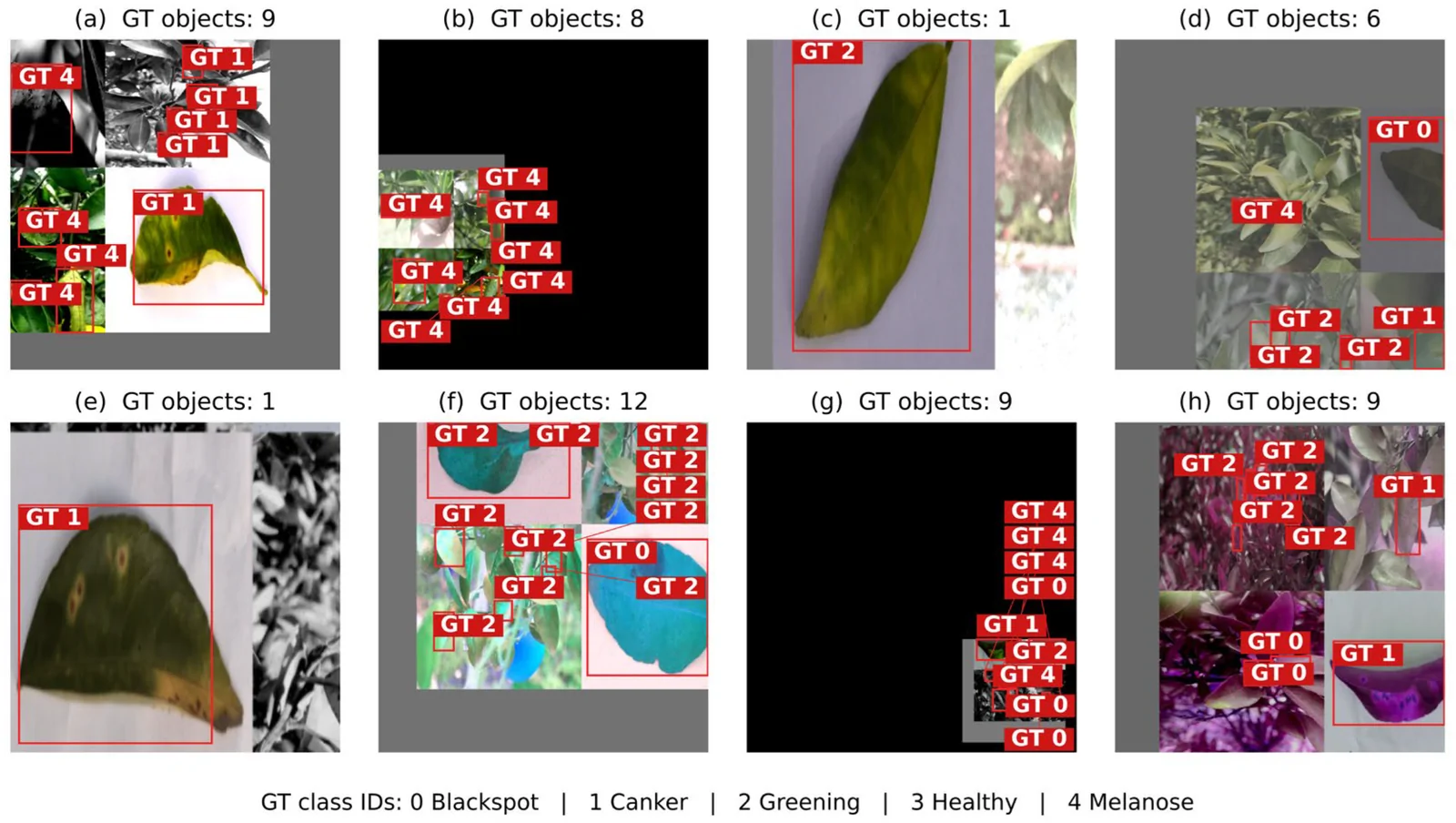
Representative training samples after augmentation, with the corresponding ground-truth bounding boxes and class labels.

At zero-based epoch 250, Mosaic, Mixup, random photometric distortion, RandomZoomOut, and RandomIoUCrop are disabled. Horizontal flipping, image resizing, tensor conversion, and one-pixel bounding-box sanitization remain active. The detector’s training forward pass then samples the configured multi-scale sizes of 480, 512, 544, 576, 608, 640, 640, 640, 672, 704, 736, 768, and 800 pixels. Validation and test inputs use resizing, tensor conversion, and normalized cxcywh box encoding. The augmentation examples in Figure 2 represent training-sample construction. Quantitative results use the fixed test evaluation described in Section 2.1, with deterministic evaluation preprocessing.

### 2.3. Detector Design and Training Rationale

**Figure 3:**
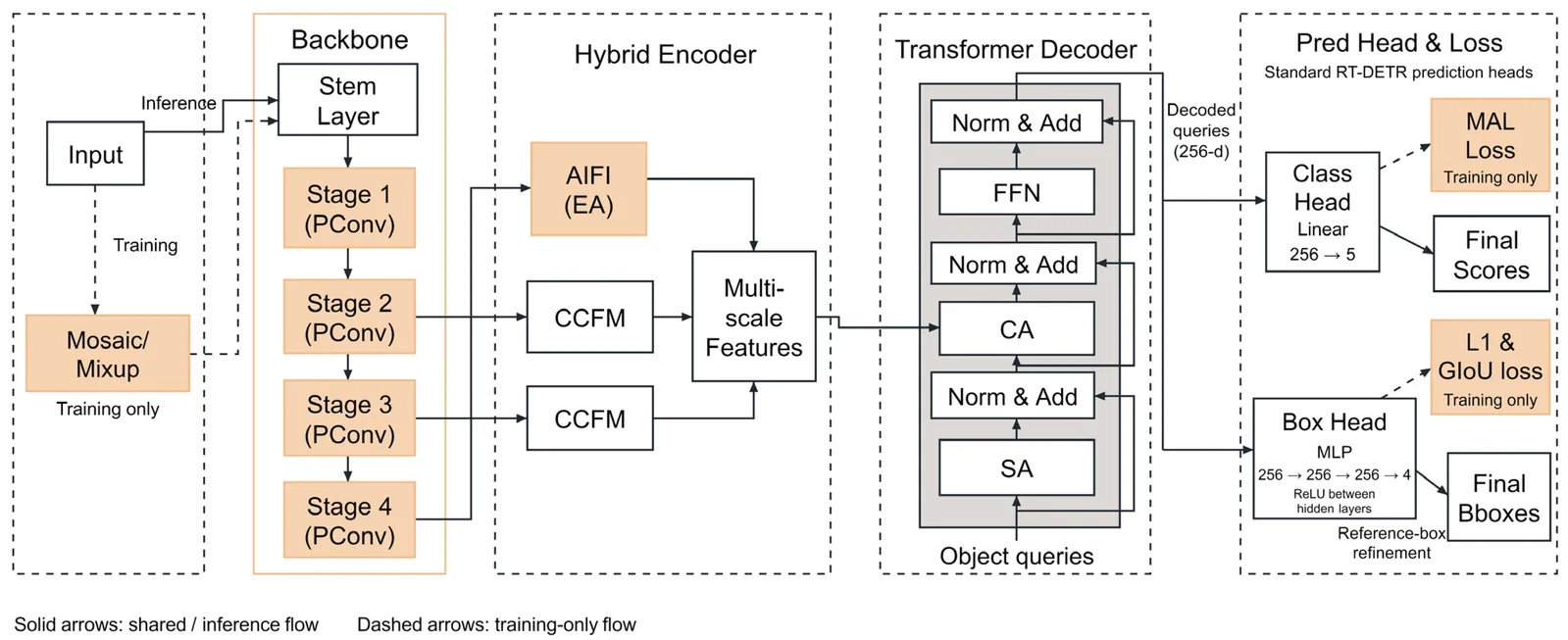
Lightweight RT-DETR architecture with the standard classification and box-regression heads. Solid arrows indicate shared or inference data flow, and dashed arrows indicate training-only data flow. Mosaic, Mixup, and loss computation apply only during training.

Figure 3 shows the backbone, hybrid encoder, and Transformer decoder. To reduce inference cost, spatial convolution is reduced in backbone Stages 1–4, and selected encoder self-attention operations use a compact context mechanism. Matching-aware supervision addresses accuracy retention during training, without adding an inference branch. The detector accepts 640 × 640 RGB images and returns class-labeled bounding boxes for the five dataset categories. The decoder retains the standard RT-DETR prediction heads [15]. The classification head maps each 256-dimensional query to five class logits. The box head uses a three-layer MLP with dimensions 256 → 256 → 256 → 4 and ReLU activations between its hidden layers, followed by reference-box refinement. These heads remain trainable; their separation is not a proposed compression mechanism.

#### 2.3.1. Efficient Spatial Feature Extraction

Partial convolution (PConv) [20] reduces spatial feature-processing cost by convolving a subset of input channels and passing the others through an identity path before fusion. Let *X* have dimensions *C* × *H* × *W*, with kernel size *k* and *C_p_* processed channels. The remaining *C* − *C_p_* channels bypass this spatial convolution operation. With equal input and output channel counts and unchanged spatial dimensions, the leading convolutional operation counts are:

(1) $$\begin{array}{c} {\mathrm{MAC}}_{\mathrm{Conv}}\;=\;H\;W\;k^{2}\;C^{2}. \end{array}$$

(2) $$\begin{array}{c} {\mathrm{MAC}}_{\mathrm{PConv}}=H\;W\;k^{2}\;C_{p}^{2}. \end{array}$$

**Figure 4:**
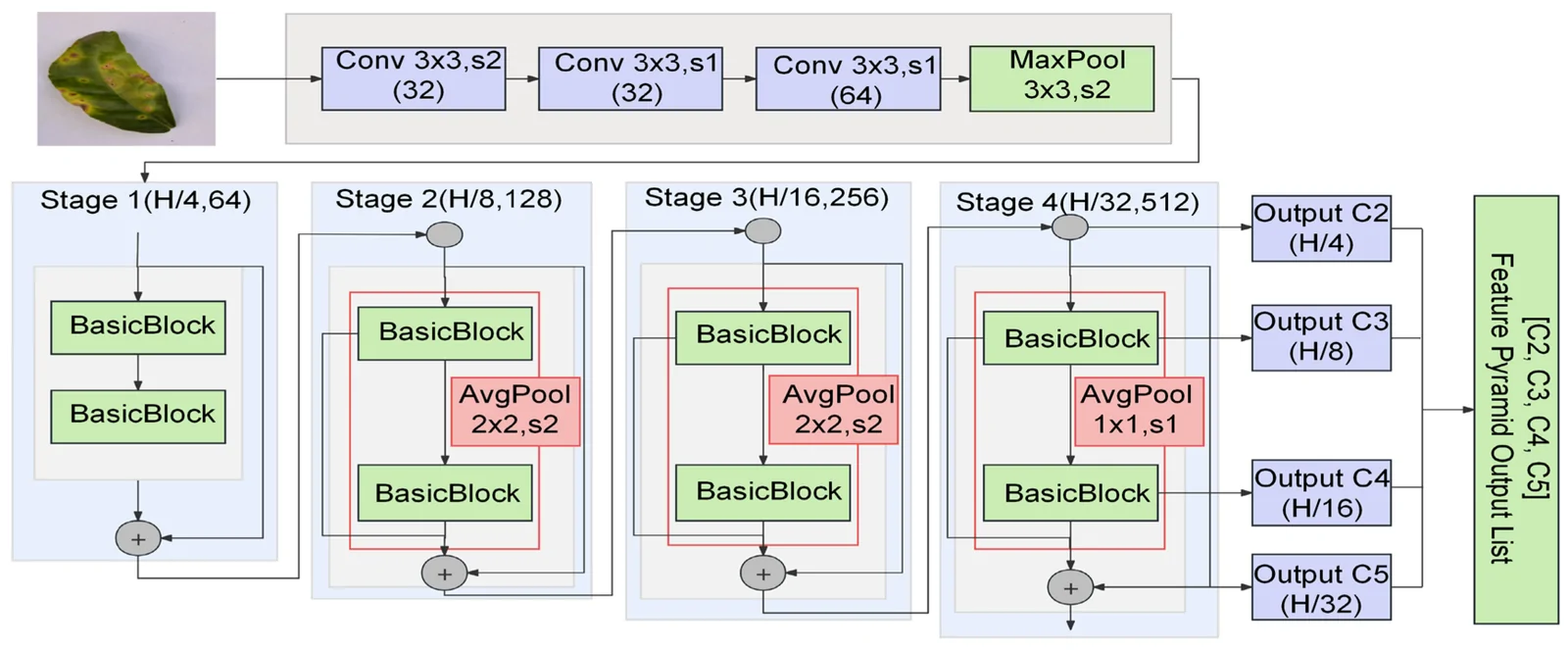
Original RT-DETR backbone architecture.

**Figure 5:**
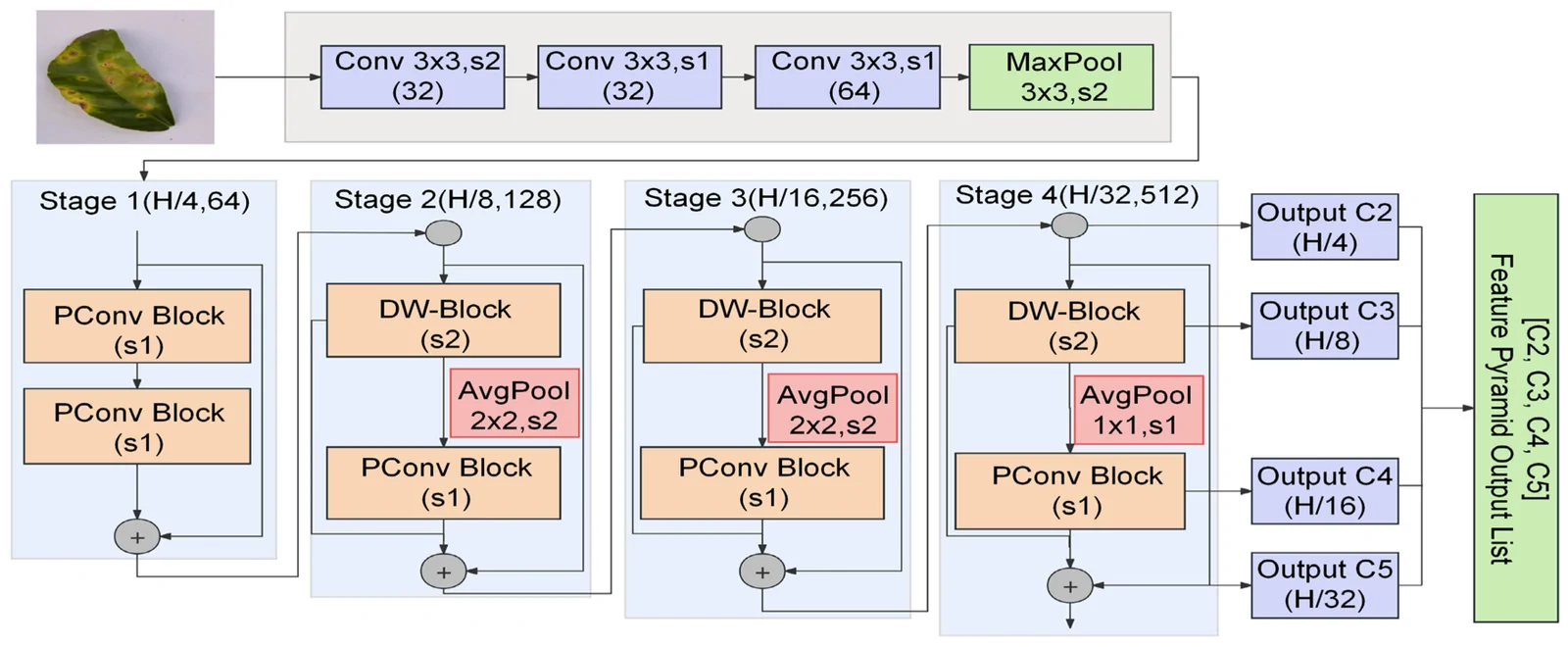
Backbone architecture with PConv-based feature extraction.

For *r* = *C*_p_/*C* = 1/4, the processed spatial-convolution term is one-sixteenth of its full-channel counterpart. Equations (1) and (2) count each multiply–accumulate as one operation; counting multiplication and addition separately doubles both terms without changing this ratio. Channel mixing and other network operations are measured in the complete-detector profiles in Table 1. Figure 4 and Figure 5 compare the original ResNet18-vd backbone and its PConv-based replacement.

As illustrated in Figure 4 and Figure 5, both backbone diagrams retain the same four-stage feature hierarchy, with spatial resolutions of *H*/4, *H*/8, *H*/16, and *H*/32 from Stages 1 to 4. The PConv-based design therefore changes feature processing within this hierarchy while preserving its multi-scale organization. The comparison in Table 1 concerns the complete backbone replacement shown in Figure 5, including the stage-transition blocks; Equations (1) and (2) describe only the processed spatial-convolution term.

#### 2.3.2. Training Supervision for the Compact Detector

**Figure 6:**
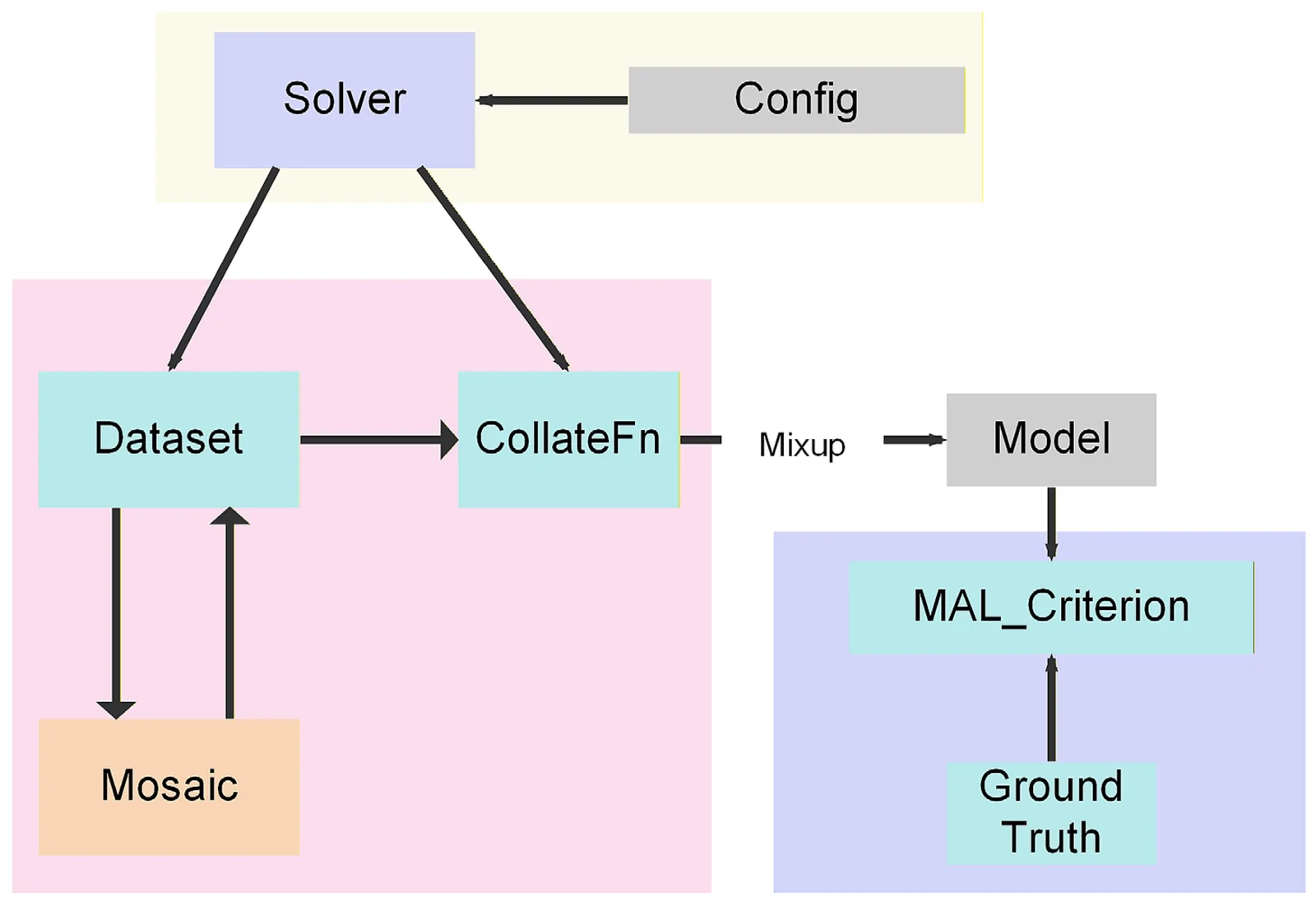
Training workflow for DOMS and matchability-aware supervision.

To strengthen training of the compact architecture, CoMATE-DETR adopts the Dense O2O Matching Strategy (DOMS) and Matchability-Aware Loss (MAL) from DEIM [22]. DOMS increases training target density through the image-composition schedule in Section 2.2 while preserving one-to-one assignment for each augmented target. MAL supplies matching-quality-aware classification supervision. Figure 6 shows how the schedule and loss train the compact detector.

A Solver controller passes the current epoch to the data loader. Mosaic and Mixup operate during early training to increase sample diversity and supervision density. When the preset stop_epoch threshold is reached, these augmentations are disabled for the remaining training epochs.

MAL links classification confidence to localization quality through a quality-aware classification loss. For a matched positive query, box IoU raised to a focusing exponent supplies the soft classification target. Unmatched predictions receive weights based on their predicted probability raised to the same exponent. This formulation retains a training signal for low-quality matches while incorporating localization quality into classification supervision [22]. DOMS and MAL operate during training and preserve the inference graph.

The ablation analysis treats DOMS and MAL as separate factors. On the fixed PConv + EA architecture, the four configurations enable neither factor, DOMS alone, MAL alone, or both factors. This arrangement quantifies the accuracy change from each training component and their combination. PConv-only and EA-only configurations assess the individual architectural modules. Training-only switches leave the learned inference structure unchanged.

#### 2.3.3. Compact Context Aggregation

**Figure 7:**
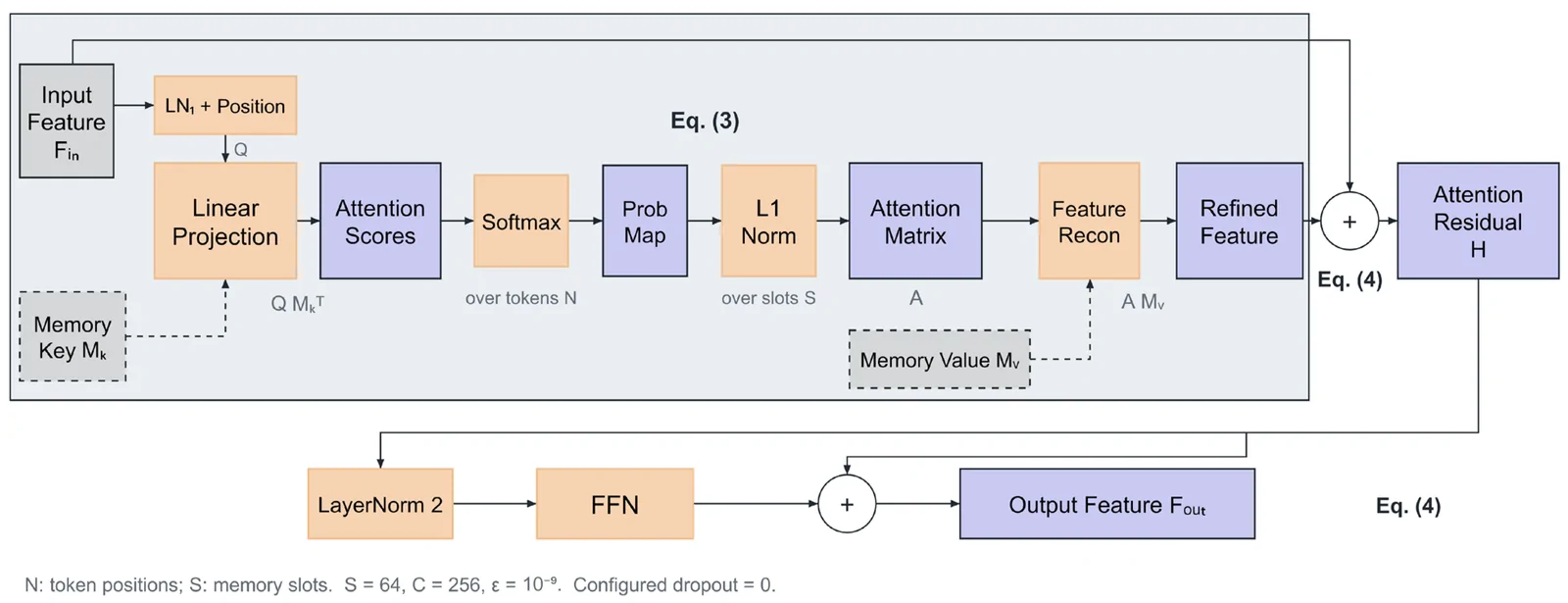
External-attention encoder layer with learnable memory key and value matrices. Equation (3) identifies query preparation and attention normalization, and Equation (4) identifies the attention residual *H* and the feed-forward residual producing *F*_out_. *S* = 64, *C* = 256, and *ε* = 10^−9^; the configured dropout rate is zero.

Context aggregation is the second inference-time design choice. Standard encoder self-attention has quadratic complexity in sequence length *N*. External attention (EA) [21] replaces selected self-attention operations with learnable memory key and value matrices, *M*_k_ and *M*_v_ (Figure 7). For input *F*_in_ with shape *B* × *N* × *C*, the pre-normalized encoder first forms *Q* by adding positional encoding *P*_pos_ to LN_1_(*F*_in_). Projection onto *M*_k_ produces attention scores. Softmax across token positions *N* followed by L1 normalization across memory slots *S* gives the normalized attention map:

(3) $$\begin{array}{c} Q={\mathrm{LN}}_{1}(F_{\mathrm{in}})+P_{\mathrm{pos}},\\ A={L1}_{S}({\mathrm{Softmax}}_{N}(Q{M_{k}}^{T})). \end{array}$$

Here, *S* is the number of memory slots (*S* = 64), and *M*_k_ and *M*_v_ are represented as *S* × *C* matrices. L1 normalization divides each token row by its sum across memory slots plus *ε* = 10^−9^. The normalized attention map *A* is projected through *M*_v_ and combined with the input to form *H*. A second pre-normalized feed-forward block and residual addition produce the encoder-layer output:

(4) $$\begin{array}{c} H\;=\;F_{\mathrm{in}}\;+\;\mathrm{Dropout}(AM_{v}),\\ F_{\mathrm{out}}\;=\;H\;+\;\mathrm{Dropout}(\mathrm{FFN}({\mathrm{LN}}_{2}(H))). \end{array}$$

For fixed memory size *S*, this formulation reduces attention complexity from O(*N*^2^*C*) to O(*NSC*). Shared memory provides global context at a lower theoretical attention cost when *S* is much smaller than *N*.

### 2.4. Experimental Setup

Experiments used Ubuntu 22.04, PyTorch 2.0.1, an Intel Xeon Platinum 8352V CPU (Intel Corporation, Santa Clara, CA, USA), and an NVIDIA GeForce RTX 4090 GPU with 24 GB of memory (NVIDIA Corporation, Santa Clara, CA, USA). The updated accuracy evaluation uses the fixed test set in Section 2.1. Reported model profiles use a 640 × 640 input on the RTX 4090. A dedicated timing mode uses a synthetic input tensor of shape 1 × 3 × 640 × 640, with each model in evaluation mode. After 50 warm-up forward passes, 200 timed forward passes are executed under torch.no_grad(), with CUDA synchronization immediately before and after timing. FPS is the reciprocal of mean forward-pass latency; preprocessing, host-to-device transfer, and post-processing are excluded. The retained RT-DETR environment contains Python 3.10.18 and THOP 0.1.1.post2209072238; archived runtime records identify CUDA 11.7.

For the detector-comparison experiments, the common training configuration used Adam with an initial learning rate of 1 × 10^−4^, a training batch size of 12, and 300 training epochs. Data augmentation was restricted to the training subset and was applied under the same experimental setting across the compared detectors. Architecture-specific network components and loss functions were retained according to the corresponding model implementations, while the dataset partition, input resolution, training duration, computing platform, and evaluation procedure were kept consistent. For the three-seed accuracy results, each run was evaluated using its final epoch-299 EMA checkpoint on the same fixed test evaluation set. Table 1 reports the arithmetic mean and sample standard deviation across seeds 0, 1, and 2; the standard deviation uses *n* − 1 = 2 in its denominator. Table 2 retains the single-value comparison profiles for the representative detectors and reports the proposed model’s three-seed mean without repeating its standard deviation. Table 3 reports a separate single evaluation with class-wise metrics.

### 2.5. Evaluation Metrics

Computational efficiency was evaluated using parameter count, floating-point operations (FLOPs), and frames per second (FPS). Detection accuracy was assessed using precision, recall, average precision (AP), and mAP@50:95.

Efficiency metrics were defined as follows:

- **Parameters (Params):** The total number of learnable weights, reported in millions (M).
- **Floating-point operations (FLOPs):** The cost of one forward pass, reported in billions (G). Values were obtained with the profile function of THOP 0.1.1.post2209072238 using a batch-one 640 × 640 input; the same default convention, with one multiply–accumulate counted as one operation, was used for every model.
- **Frames per second (FPS):** The reciprocal of the mean timed forward-pass latency for batch size one after warm-up; preprocessing, data transfer, and post-processing are excluded. In Equation (5), *T*_avg_ is measured in seconds per image:

(5) $$\begin{array}{c} \mathrm{FPS}=\frac{1}{T_{\mathrm{avg}}}. \end{array}$$

Detection accuracy followed the MS COCO protocol [26]. Precision (*P*) and recall (*R*) are:

(6) $$\begin{array}{c} P\;=\;\frac{\mathrm{TP}}{\mathrm{TP}\;+\;\mathrm{FP}},\;R\;=\;\frac{\mathrm{TP}}{\mathrm{TP}\;+\;\mathrm{FN}}. \end{array}$$

Average precision (AP) summarizes the interpolated precision–recall curve. The integral below gives the area interpretation; the COCO evaluator implements AP as the mean interpolated precision at 101 recall thresholds from 0 to 1:

(7) $$\begin{array}{c} \mathrm{AP}\;=\;\int_{0}^{1}\;P(R)\;dR. \end{array}$$

mAP@50:95 averages AP across 10 IoU thresholds from 0.50 to 0.95 in steps of 0.05 and across all five classes:

(8) $$\begin{array}{c} {\mathrm{mAP}}_{50:95}\;=\;\frac{1}{10}\;\sum_{k\;=\;0}^{9}\;{\mathrm{mAP}}_{\mathrm{IoU}\;=\;0.50\;+\;0.05k}. \end{array}$$

Parameter and operation reductions are calculated as the difference from the stated reference divided by that reference. Accuracy changes are reported in percentage points, while throughput changes are relative percentages. These distinctions prevent a change in the comparison denominator from being interpreted as a different measurement. Percentages derived from tabulated values are subject to the rounding shown in the Tables.

## 3. Results

### 3.1. Accuracy and Computational Cost Under Ablation

Table 1 separates architectural compression from DOMS and MAL supervision. Exp. 1 is the compatible baseline, Exp. 2 uses PConv alone, and Exp. 3 denotes EA alone. Exps. 4–7 form the DOMS/MAL factorial comparison on the fixed PConv + EA architecture. Exp. 6 is the complete CoMATE-DETR model.

The compatible baseline achieved 91.79 ± 0.41% mAP@50:95, and PConv alone achieved 91.09 ± 0.12%, a decrease of 0.70 percentage points. EA alone achieved 90.62 ± 0.20% with 19.8 M parameters. PConv + EA with both training factors disabled achieved 90.56 ± 0.31%. Enabling DOMS alone increased this mean to 91.36 ± 0.27%, while enabling MAL alone increased it to 92.94 ± 0.89%. The complete configuration achieved 91.70 ± 0.41%, an increase of 1.14 percentage points over PConv + EA and 0.61 percentage points over PConv alone.

The complete model combines 91.70 ± 0.41% mAP@50:95 with a reported profile of 9.7 M parameters, 17.6 GFLOPs, and 106 FPS. Relative to the 20.1 M-parameter, 30.2-GFLOP baseline, parameter count and operation count decrease by 51.7% and 41.7%, respectively. Its mean AP is 0.09 percentage points below the compatible baseline, while the reported forward-pass throughput increases from 94 to 106 FPS.

The separate supervision controls show that MAL increases mean AP by 2.38 percentage points with DOMS disabled and by 0.34 percentage points with DOMS enabled. DOMS increases mean AP by 0.80 percentage points with MAL disabled; with MAL enabled, the mean changes from 92.94% to 91.70%. The PConv + EA + MAL configuration therefore provides the highest measured AP among the completed ablations, while Exp. 6 reports the complete four-component design.

### 3.2. Training Process and Convergence Analysis

**Figure 8:**
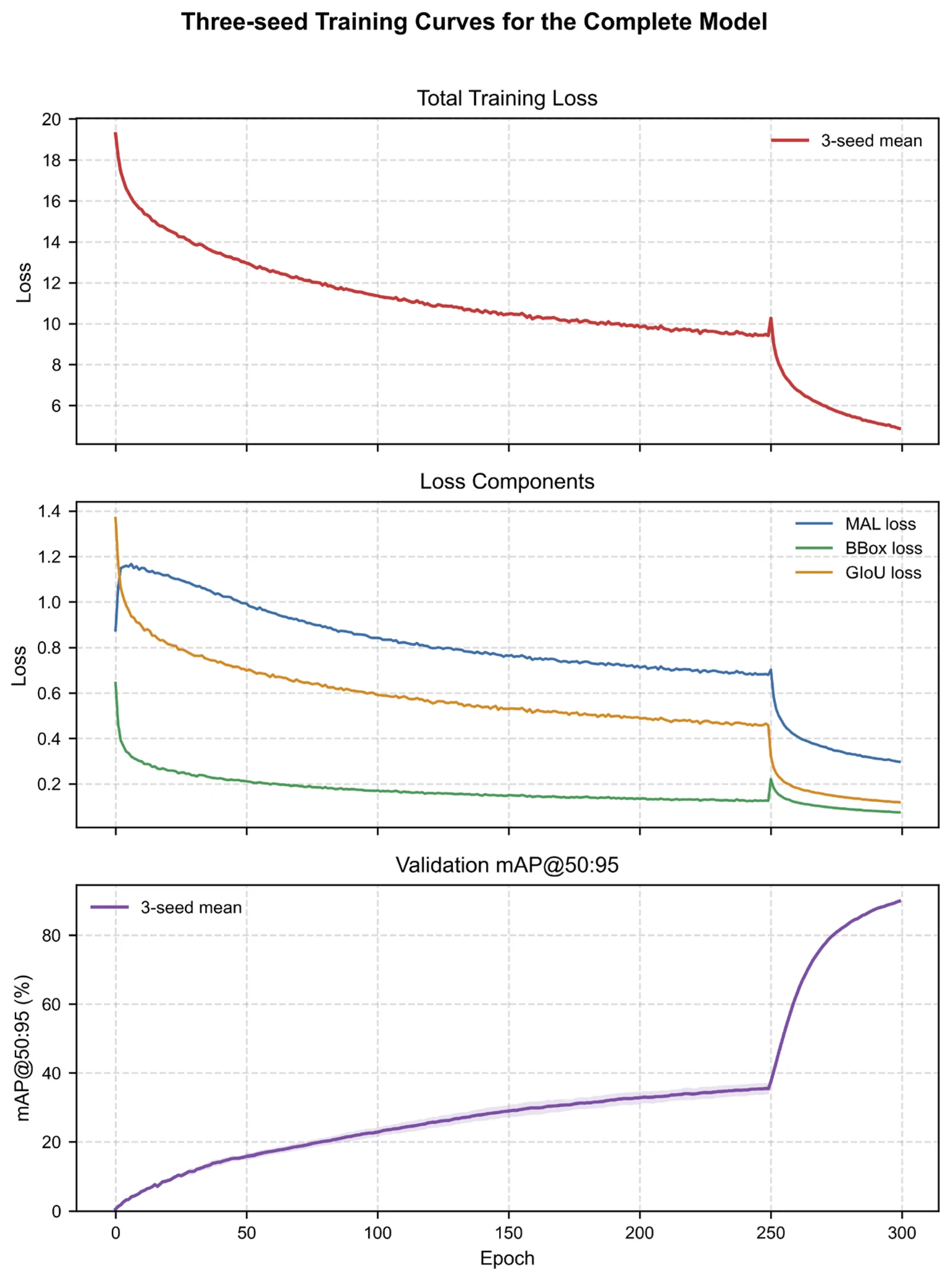
Three-seed mean training losses and validation mAP@50:95 over 300 epochs in three vertically stacked panels. From top to bottom: total training loss; MAL, bounding-box, and GIoU losses; and validation mAP@50:95. The validation panel shows the mean curve with the three-seed standard-deviation band.

Figure 8 presents three-seed mean training curves over 300 epochs in three vertically stacked panels: (i) total training loss; (ii) MAL, bounding-box, and GIoU loss components; and (iii) validation mAP@50:95. Total loss and the component losses decrease rapidly during early training before approaching stable plateaus. The epoch-250 augmentation transition is followed by a sharper rise in validation mAP toward 90%. The curves summarize training behavior; aggregate test results are reported in Table 1.

### 3.3. Accuracy and Throughput Comparison

**Figure 9:**
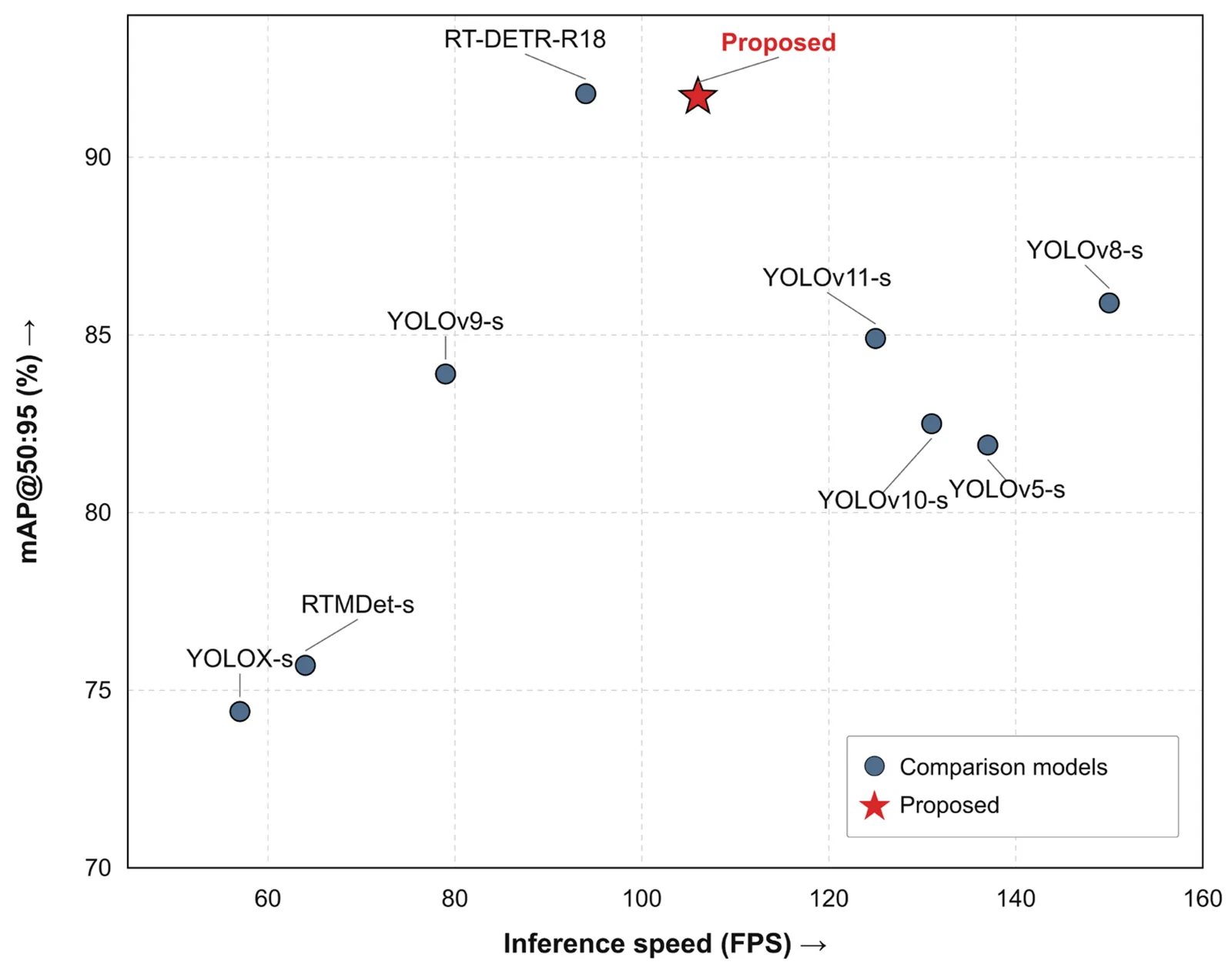
Reported speed–accuracy trade-off for CoMATE-DETR and representative real-time object detectors. The points use the mAP@50:95 values listed in Table 2. Arrows indicate increasing FPS and mAP; higher values on both axes are preferable.

Table 2 summarizes the reported parameter counts, operation counts, throughput, and mAP@50:95 values of representative real-time detectors [15,16,17,18,19,27,28,29]. Figure 9 visualizes the reported speed–accuracy positions for the comparison models and the proposed detector.

In this comparison Table, CoMATE-DETR is listed at 91.70% mAP@50:95, compared with 91.79% for the RT-DETR-R18 comparison profile. The proposed detector is 0.09 percentage points lower than this profile, while reducing the parameter count from 20.1 M to 9.7 M and the operation count from 30.2 to 17.6 GFLOPs. Its reported 17.6 GFLOPs are also 18.9% below the YOLOv11-s profile.

Figure 9 places the proposed detector at 106 FPS and 91.70% mAP@50:95, alongside the RT-DETR-R18 comparison profile at 94 FPS and 91.79%; the other reported comparison profiles are shown as well. The plot summarizes the speed–accuracy positions listed in Table 2.

Operation count and measured throughput describe different aspects of model efficiency. The reported YOLOX-s and RTMDet-s profiles contain fewer operations than CoMATE-DETR, while their measured throughput is lower. YOLOv8-s has a higher operation count and higher throughput. These measurements show the importance of reporting both architecture cost and execution speed under the specified hardware and software conditions.

### 3.4. Per-Class Detection Performance

Table 3 reports class-wise AP values from a single evaluation on the fixed 566-entry test set described in Section 2.1. The Table complements the three-seed aggregate results in Table 1 with per-class AP50:95, AP50, and AP75.

In this single evaluation, mean AP50:95, AP50, and AP75 were 92.06%, 99.16%, and 97.49%, respectively.

The five-category mean assigns equal weight to Healthy and each disease category. AP summarizes the precision–recall relationship across confidence thresholds and localization requirements. Healthy achieved the highest AP50:95 at 97.20%, while Canker achieved 88.36%, a difference of 8.84 percentage points. AP50 ranged from 98.71% for Canker to 99.85% for Healthy.

### 3.5. External Cross-Dataset Evaluation

The external evaluation used BDLemonLeaf V6, a Mendeley Data package of lemon-leaf images collected at multiple sites in Dhaka, Bangladesh [30]. The matched Greening subset contained 597 images and 597 leaf-level bounding boxes. A review of the external and primary image lists found no identical images in common. The frozen seed-2 epoch-299 EMA checkpoint was evaluated with deterministic 640 × 640 preprocessing. The external package was used only for evaluation; no retraining, fine-tuning, threshold adjustment, or external-data model selection was performed. The matched Greening leaf-detection results are summarized in Table 4.

CoMATE-DETR obtained 63.05% AP50:95, 95.32% AP50, and 77.67% AP75 on the external Greening subset. This result provides a cross-dataset measurement of transfer under the fixed checkpoint and preprocessing protocol and complements the five-category results in Table 1, Table 2 and Table 3.

### 3.6. Visualization of Detection Results

**Figure 10:**
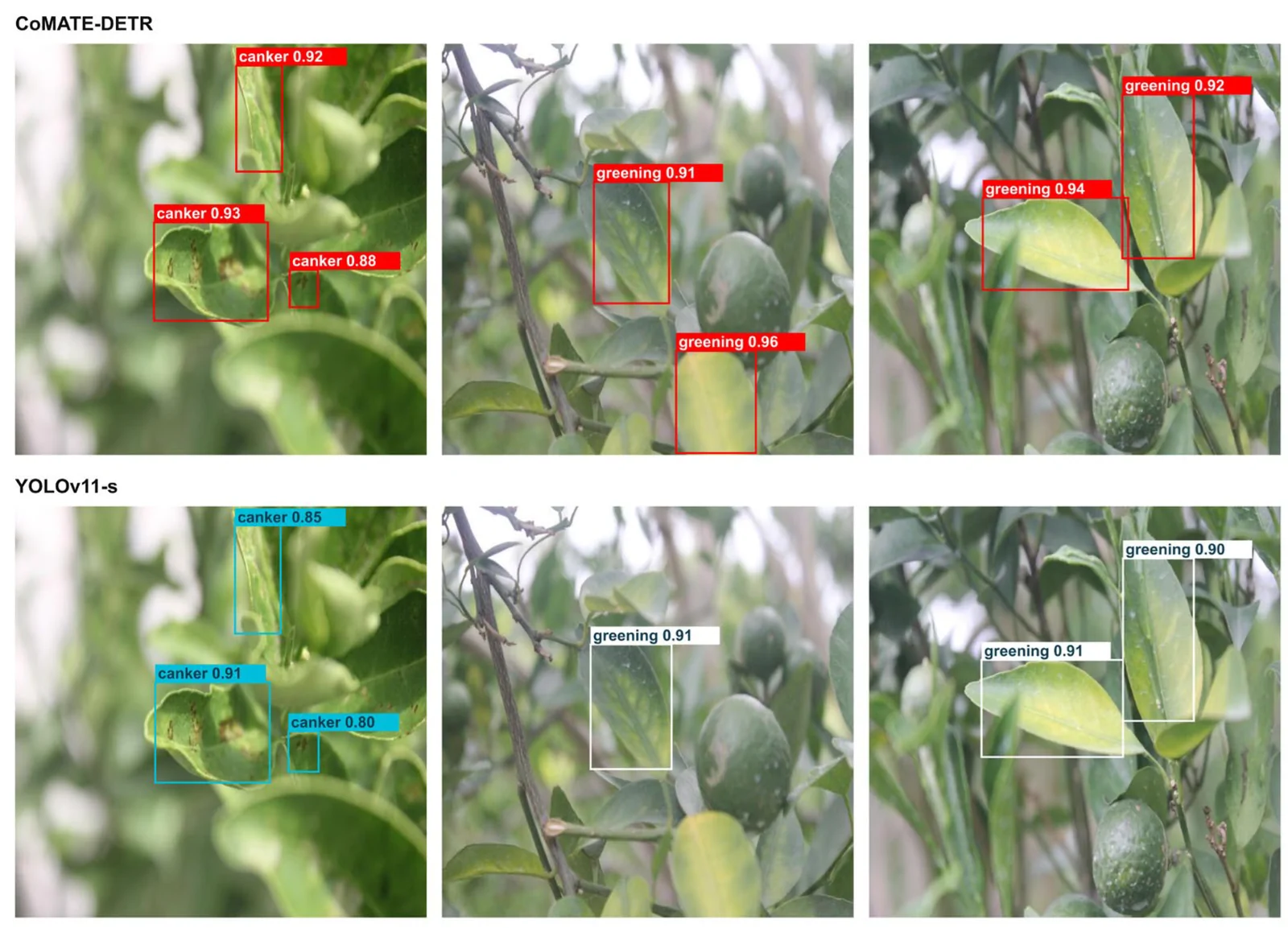
Qualitative detection examples for the proposed lightweight RT-DETR (top row, red boxes) and YOLOv11-s (bottom row, white and blue boxes). Only complete, readable in-frame annotations are displayed; boundary-touching boxes and truncated labels are omitted. Displayed confidence scores are model-specific.

Figure 10 presents qualitative detection examples for CoMATE-DETR and YOLOv11-s. Red boxes denote CoMATE-DETR, while white and blue boxes denote YOLOv11-s. The images display predicted locations, labels, and model-specific confidence scores. The updated quantitative accuracy is reported in Table 1, Table 2 and Table 3.

### 3.7. Qualitative Assessment of Additional Web Images

**Figure 11:**
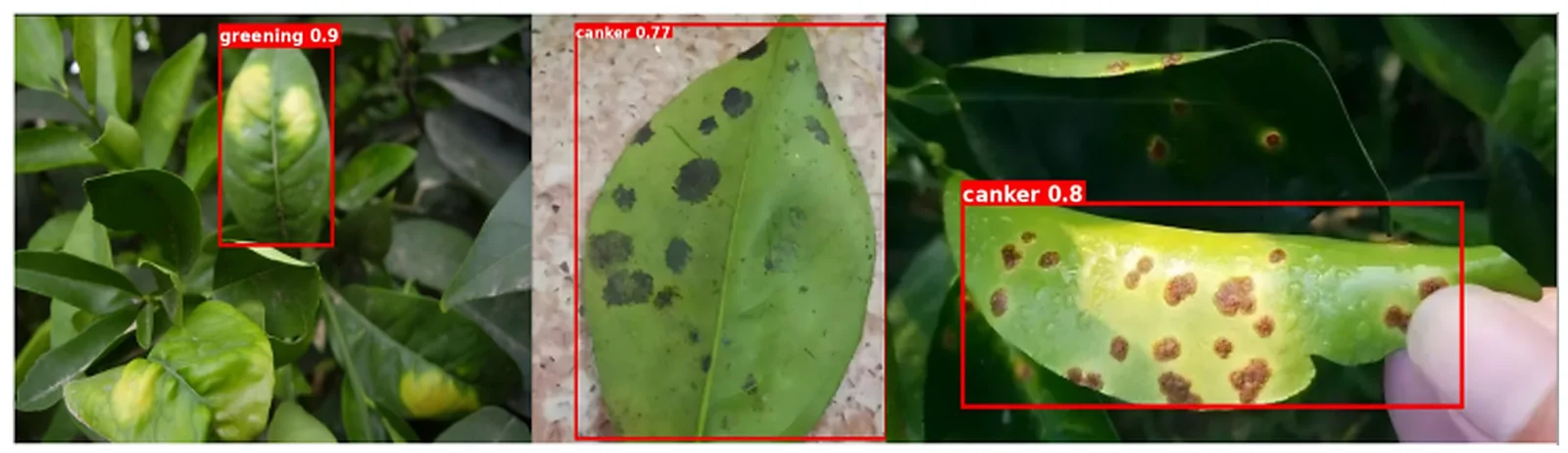
Illustrative model predictions on additional unannotated web images of citrus leaves.

The trained model was also applied to additional publicly accessible images of diseased citrus leaves (Figure 11). These examples cover variation in illumination, viewing angle, background, and symptom appearance. They were used for qualitative visualization only, separate from model training and the quantitative evaluation reported in the Tables.

The predicted boxes illustrate the behavior of the detector on the selected images and complement the dataset-based evaluation. These unannotated examples provide visual inspection of model outputs alongside the quantitative results.

## 4. Discussion

The main finding is that CoMATE-DETR achieves a compact architecture and high detection AP through coordinated architecture and training design. The three-seed evaluation gives 91.70 ± 0.41% mAP@50:95 for the complete model and 91.79 ± 0.41% for the compatible baseline, alongside substantial reductions in parameters and operation count. The factorial ablations quantify the separate effects of DOMS and MAL on the compressed architecture.

### 4.1. Accuracy Recovery After Backbone Compression

PConv [20] reduces feature-extraction cost, and the supervision controls quantify accuracy recovery on the compact architecture. PConv + EA reaches 90.56 ± 0.31% mAP@50:95 with both DOMS and MAL disabled. The complete training configuration reaches 91.70 ± 0.41%, a gain of 1.14 percentage points. PConv + EA + MAL reaches 92.94 ± 0.89%, the highest mean among the completed ablations. The separate controls therefore identify MAL as the strongest accuracy-improving training component under this evaluation protocol [22].

### 4.2. Selecting the Accuracy and Cost Trade-Off

The architecture and supervision choices define distinct operating points. PConv alone yields 91.09 ± 0.12% mAP@50:95 with 10.0 M parameters, and EA alone yields 90.62 ± 0.20% with 19.8 M parameters. PConv + EA without DOMS or MAL yields 90.56 ± 0.31% with 9.7 M parameters. On that fixed architecture, PConv + EA + MAL gives 92.94 ± 0.89%, and the complete DOMS + MAL configuration gives 91.70 ± 0.41%. This comparison locates the highest-AP training configuration and quantifies the full model’s accuracy and model-size profile [21].

### 4.3. Implications for Vision-Based Sensing

Camera-based citrus monitoring requires an image-analysis component that can localize and classify visual symptoms at manageable computational cost. Earlier sensing studies [6,7,8,9] demonstrate the relevance of such components to mobile, canopy-level, and networked monitoring workflows. The present model addresses this computational component by reducing parameters and FLOPs while retaining high AP in the evaluated image collection. Its compact architecture provides a basis for subsequent integration with acquisition and decision-support systems. System-level latency also includes image capture, preprocessing, data transfer, and output handling, and should be distinguished from detector throughput.

The three-seed results quantify training-seed variation on the fixed evaluation set, and the reported profiles characterize model-forward execution on the RTX 4090. The BDLemonLeaf evaluation adds a cross-dataset Greening measurement using the frozen checkpoint. Together, these results report in-domain and cross-dataset detector performance under the specified protocols.

The ablations distinguish the roles of architectural modules and training supervision. PConv reduces spatial computation in the backbone, EA provides compact context aggregation, and DOMS and MAL modify training. On the fixed PConv + EA architecture, the MAL gain is 2.38 percentage points without DOMS and 0.34 percentage points with DOMS. The DOMS effect is +0.80 percentage points without MAL and −1.24 percentage points with MAL. These measured differences establish the configuration-dependent interaction between the two training factors.

Representative real-time detectors use different combinations of prediction heads, assignment rules, feature aggregation, and post-processing. CoMATE-DETR retains the RT-DETR set-prediction decoder and concentrates compression in spatial processing and encoder context aggregation. The updated accuracy comparison with the compatible baseline gives a decrease of 0.09 percentage points in mean AP together with a 51.7% parameter reduction and a 41.7% operation-count reduction. This result quantifies the detector’s accuracy–efficiency balance.

### 4.4. Evaluation Scope

The primary evaluation uses the fixed citrus image collection and the sample weighting specified in Section 2.1. The BDLemonLeaf V6 evaluation measures transfer on 597 Greening images with leaf-level boxes. The external and primary image lists were reviewed separately, and the external result is reported for the matched Greening task, while the five-category results remain those in Table 1, Table 2 and Table 3.

## 5. Conclusions

This study developed CoMATE-DETR for RGB-image detection of four citrus leaf disease categories and a healthy category. Across three training seeds, the complete detector achieved 91.70 ± 0.41% mAP@50:95. Its reported profile contains 9.7 M parameters, 17.6 GFLOPs, and a throughput of 106 FPS on an RTX 4090. Compared with the compatible baseline’s 91.79 ± 0.41% mAP@50:95, the complete detector reduces parameter count by 51.7% and operation count by 41.7%, with a mean AP difference of −0.09 percentage points. Matching-aware training increases AP from 90.56% for PConv + EA to 91.70% for the full training configuration. The external BDLemonLeaf V6 Greening evaluation achieved 63.05% AP50:95, 95.32% AP50, and 77.67% AP75 with the frozen checkpoint.

The factorial analysis identifies PConv + EA + MAL as the highest-AP completed configuration, achieving 92.94 ± 0.89% mAP@50:95. The results distinguish the individual and combined effects of DOMS and MAL and provide concrete configurations for selecting accuracy and computational cost. CoMATE-DETR provides a compact image-analysis foundation for camera-based citrus monitoring.

## Figures and Tables

**Table 1:** Three-seed detection accuracy and computational cost under ablation.

No.	Modules				Metrics			
	PConv	DOMS	EA	MAL	Params (M)	FLOPs (G)	mAP@50:95 (%)	FPS
1					20.1	30.2	91.79 ± 0.41	94
2	✓				10.0	17.6	91.09 ± 0.12	100
3			✓		19.8	30.2	90.62 ± 0.20	94
4	✓		✓		9.7	17.6	90.56 ± 0.31	100
5	✓	✓	✓		9.7	17.6	91.36 ± 0.27	104
**6**	**✓**	**✓**	**✓**	**✓**	**9.7**	**17.6**	**91.70 ± 0.41**	**106**
7	✓		✓	✓	9.7	17.6	92.94 ± 0.89	100

Note: ✓ indicates that the corresponding module is enabled; a blank cell indicates that it is disabled.

**Table 2:** Comparison with representative real-time object detectors.

Model	Params (M) ↓	FLOPs (G) ↓	FPS ↑	mAP@50:95 (%) ↑
YOLOv5-s	9.1	24.2	137	81.9
YOLOv8-s	11.1	28.8	150	85.9
YOLOv9-s	7.2	27.6	79	83.9
YOLOv10-s	8.1	25.1	131	82.5
YOLOv11-s	9.4	21.7	125	84.9
YOLOX-s	8.9	13.3	57	74.4
RTMDet-s	8.8	14.7	64	75.7
RT-DETR-R18	20.1	30.2	94	91.79
**Proposed**	**9.7**	**17.6**	**106**	91.70

Note: ↓ indicates that lower values are preferable, and ↑ indicates that higher values are preferable.

**Table 3:** Per-class detection performance from a single test evaluation.

Category	AP50:95 (%)	AP50 (%)	AP75 (%)
Blackspot	91.32	98.81	97.84
Canker	88.36	98.71	96.14
Greening	90.90	99.65	96.87
Healthy	97.20	99.85	97.83
Melanose	92.51	98.77	98.77
**Mean**	**92.06**	**99.16**	**97.49**

Note: Category names follow the dataset labels. AP50:95 averages AP over IoU thresholds from 0.50 to 0.95 in increments of 0.05. The Mean row is the unweighted average across five categories. Values are from one evaluation of the fixed 566-entry test set.

**Table 4:** External cross-dataset evaluation on the matched Greening leaf-detection task.

External Subset	Images	Valid Boxes	AP50:95 (%)	AP50 (%)	AP75 (%)
BDLemonLeaf V6 Greening	597	597	63.05	95.32	77.67

Note: AP50:95 averages AP over IoU thresholds from 0.50 to 0.95 in increments of 0.05. The values are calculated for the matched Greening leaf-detection task and are reported separately from the five-category mAP.

## Data Availability

The primary Jiangxi Citrus Leaf Disease Dataset is described by Wu et al. [24] and was obtained through a third-party online data platform. It was not collected by the authors and is not presented here as an official public benchmark. The original images are not redistributed because of third-party access and redistribution conditions. The external BDLemonLeaf V6 package is available from Mendeley Data [30]. The audited class definitions, split configuration, image lists, annotation statistics, and external evaluation package are available from the corresponding author upon reasonable request.

## Appendix A. Dataset Split and Annotation Statistics

The following Tables document the curated split inventory and annotation cleanup. Image counts report the file entries in each split. Valid-box counts exclude the six zero-area records.

**Table A1:** Image and valid-instance counts by split and object size.

Split	Images	Valid Boxes	Small	Medium	Large
Training	4526	10,165	186	3184	6795
Validation	566	1455	53	533	869
Test	566	1151	19	290	842
**Total**	**5658**	**12** **,** **771**	**258**	**4007**	**8506**

Note: Object sizes use original-image box area *A*: small, 0 < *A* < 1024 pixels^2^; medium, 1024 ≤ *A* < 9216 pixels^2^; large, *A* ≥ 9216 pixels^2^.

**Table A2:** Class-wise image and valid-instance counts for each split.

Split	Category	Images	Valid Boxes	Small	Medium	Large
Training	Blackspot	1171	2675	107	942	1626
Training	Canker	1316	2676	69	905	1702
Training	Greening	888	2213	5	956	1252
Training	Healthy	275	685	0	136	549
Training	Melanose	876	1916	5	245	1666
Validation	Blackspot	153	401	10	167	224
Validation	Canker	162	387	21	143	223
Validation	Greening	121	341	20	139	182
Validation	Healthy	33	95	0	36	59
Validation	Melanose	97	231	2	48	181
Test	Blackspot	138	288	13	76	199
Test	Canker	172	346	6	132	208
Test	Greening	101	214	0	55	159
Test	Healthy	38	74	0	3	71
Test	Melanose	117	229	0	24	205

Note: Category names reproduce the supplied labels. Healthy is class ID 3 and has instance bounding boxes for joint localization and classification.

**Table A3:** Cross-split image-list audit.

Split Pair	Identical Images
Training–validation	0
Training–test	0
Validation–test	0

Note: The audit found no completely identical image shared by any two final splits.

**Table A4:** Source-image records associated with zero-area Melanose annotations.

Source Split	Source Image	Recorded Action
Training	3956.jpg	Removed the invalid box; retained the image and its other boxes.
Training	3957.jpg	Removed the invalid box; retained the image and its other boxes.
Training	3958.jpg	Removed the invalid box; retained the image and its other boxes.
Validation	3960.jpg	Removed the affected invalid box records; retained all valid images and boxes.
Test	3955.jpg	Source record was already excluded during split preparation.
Test	3959.jpg	Removed the invalid box; retained the image and its other boxes.

Note: Invalid boxes had [x, y, width, height] = [314.25, 302, 0, 6]. The final splits lost six invalid box records: training, three; validation, two; test, one. The cleanup retained every remaining image and its other valid boxes. The original annotation backup and individual processing log are retained in the data package.
